# Imaging mRNA Expression in Live Cells via PNA*·*DNA Strand Displacement-Activated Probes

**DOI:** 10.1155/2012/962652

**Published:** 2012-09-26

**Authors:** Zhenghui Wang, Ke Zhang, Karen L. Wooley, John-Stephen Taylor

**Affiliations:** ^1^Department of Chemistry, Washington University, St. Louis, MO 63130, USA; ^2^Department of Chemistry, Texas A&M University, P.O. Box 30012, College Station, TX 77842-3012, USA

## Abstract

Probes for monitoring mRNA expression *in vivo* are of great interest for the study of biological and biomedical problems, but progress has been hampered by poor signal to noise and effective means for delivering the probes into live cells. Herein we report a PNA**·**DNA strand displacement-activated fluorescent probe that can image the expression of iNOS (inducible nitric oxide synthase) mRNA, a marker of inflammation. The probe consists of a fluorescein labeled antisense PNA annealed to a shorter DABCYL^plus^-labeled DNA which quenches the fluorescence, but when the quencher strand is displaced by the target mRNA the fluorescence is restored. DNA was used for the quencher strand to facilitate electrostatic binding of the otherwise netural PNA strand to a cationic shell crosslinked knedel-like (cSCK) nanoparticle which can deliver the PNA*·*DNA duplex probe into cells with less toxicity and greater efficiency than other transfection agents. RAW 264.7 mouse macrophage cells transfected with the iNOS PNA*·*DNA probe via the cSCK showed a 16 to 54-fold increase in average fluorescence per cell upon iNOS stimulation. The increase was 4 to 7-fold higher than that for a non-complementary probe, thereby validating the ability of a PNA*·*DNA strand displacement-activated probe to image mRNA expression *in vivo*.

## 1. Introduction

There has been great interest in developing real-time fluorescent imaging agents for mRNA expression *in vivo* that are based on antisense oligodeoxynucleotides and analogs [1–3]. There are two main problems in getting such systems to work well. The first is to deliver the agents efficiently into the cytoplasm, and the second is to minimize background signal from unbound probe. The main problem with getting nucleic acids and analogs into the cytoplasm is that they are membrane impermeable, thereby requiring the use of a physical, chemical, or biochemical device or agent [4]. Many mRNA-imaging studies have used microinjection, electroporation, or pore forming agents such as streptolysin O (SLO), but such agents would be unsuitable for *in vivo* work. Others have made use of cell-penetrating peptides, or transfection agents, but these often result in endocytosis and trapping of the probe in endosomes which reduces the amount of probes in the cytoplasm and can lead to nonspecific background signal. To reduce the background signal from unbound probe, probes have been designed to emit fluorescence only in the presence of target mRNA by a variety of strategies. Among these are molecular beacons, binary and dual FRET probes, strand-displacement probes, quenched autoligating probes, FIT-probes, and nucleic-acid-triggered probe activation [5, 6]. 

One general approach to activatable probes makes use of a fluorophore-quencher pair, typified by the molecular beacon strategy [5, 7]. Molecular beacons consist of a fluorescent molecule and a quencher that are conjugated to both ends of an antisense nucleic acid sequence which may or may not have a short complementary stem. When free in solution, the fluorophore component is quenched by either FRET, in which case the energy of the excited fluorophore is transferred to a quencher by a through space mechanism [8]; or by “contact quenching,” in which a fluorophore and a quencher are close enough that they can form a nonfluorescent complex [9]. Upon binding to the target RNA, the fluorophore and quencher are physically held apart by duplex formation, and fluorescence is restored. While this is an elegant system, it suffers from background fluorescence due to nonspecific binding events that lead to separation of the fluorophore and the quencher.

**Figure 1:**
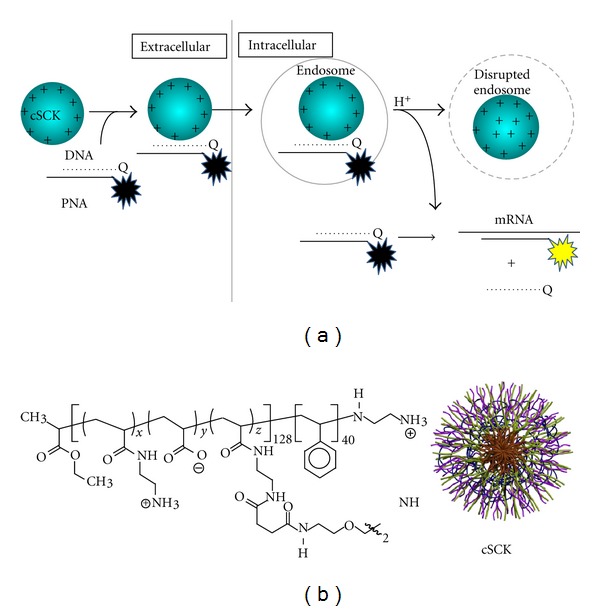
Schematic representation of cSCK-mediated delivery of strand-displacement-activated PNA·DNA probes for imaging mRNA in living cells. (a) The probes consist of a fluorescently labeled nondegradable antisense PNA (peptide nucleic acid) hybridized to a shorter negatively charged complementary DNA strand bearing a quencher, leaving a short single-stranded section of PNA (the toehold). The nonfluorescent PNA*∙*DNA duplex probe is then electrostatically bound to the cationic-shell-crosslinked knedel-like nanoparticle (cSCK). The positive nature of the cSCK facilitates its endocytosis, and the presence of unprotonated amines facilitates disruption of the endosome by the proton-sponge effect which enables the strand-displacement probe to escape into the cytoplasm. Binding of the toe-hold portion of the PNA to the target mRNA sequence then facilitates strand displacement of the quenching DNA strand by branch migration and results in restoration of fluorescence to the PNA strand. (b) Structure of the cSCK formed by crosslinking the block copolymers following micellization *x* ≈ 122, *y* ≈ 0, *z* ≈ 6.

A bimolecular version of a molecular beacon, often referred to as a strand-displacement probe, makes use of an antisense oligonucleotide conjugated to a fluorescent probe that is annealed to a shorter complementary oligonucleotide conjugated to a quencher [10–12] (Figure 1(a)). In this system, the duplex region is much longer and much more stable than the generally short duplex stem used in molecular beacons. Despite the high stability, rapid strand exchange can take place because the short section of single strand on the probe strand can hybridize to the target RNA and facilitate the thermodynamically favorable displacement of the quencher strand through branch migration. The rate of strand-displacement depends on the single-strand length (“toehold”) while the extent of reaction will depend on the difference in length between the fluorescent and quenching probes [13]. The larger the difference, the longer the un-paired section and the faster the rate for displacing the shorter strand by the target mRNA and the more complete the displacement.

While there are numerous studies using molecular beacons for imaging of gene expression *in vivo*, there have only been a few reports of the use of strand-displacement probes. Hnatowich and coworkers constructed a probe from a 25-mer phosphorodiamidate morpholino (MORF) oligomer conjugated to a Cy5.5 and a complementary 18-mer cDNA conjugated to a BHQ3 quencher. They showed that this probe could image a complementary biotinylated 25-mer MORF oligomer immobilized on streptavidin polystyrene microspheres that were intramuscularly implanted into a mouse [14]. The same group also utilized a probe consisting of a 25-mer phosphorothioate DNA bearing Cy5.5 and a 10-mer complementary ODN with the BHQ3 quencher to image the KB-G2 tumor in mice which overexpresses the multi-drug-resistant mdr1 mRNA [15]. In another approach, Mirkin and coworkers developed “nanoflares” in which antisense ODNs to a target mRNA are conjugated to a gold nanoparticle and then hybridized to a shorter strand of complementary DNA bearing Cy5 which is quenched by the gold nanoparticle. When taken up by cells containing the target mRNA, the Cy5-bearing ODN becomes displaced resulting in fluorescence activation [16]. In their design, however, the fluorescent reporter becomes displaced by the mRNA making it unable to report on the location of the mRNA within the cell. DNA-based probes also suffer from premature intracellular degradation, which generates a high background signal.

All previous studies of the strand displacement-activated probes have made use of either DNA, phosphorothioate, or phosphorodiamidate morpholino, and none have made use of PNA. PNAs have a number of properties that make them ideal for strand-displacement probe technology. They are very resistant to chemical and enzymatic degradation, bind with higher affinity to RNA than DNA, and able to invade regions of RNA with secondary structure [17, 18]. They also do not activate RNAse H degradation of the target RNA and protect a complementary ODN from degradation. We have also shown that PNA·ODN duplexes can be efficiently delivered into cells by cationic-shell-crosslinked nanoparticles (cSCKs) (Figure 1(b)) through favorable electrostatic interactions, and remain highly bioactive [19, 20]. The cSCKs are also much less cytotoxic and efficient than the commonly used Lipofectamine. 

To determine whether or not PNA·ODN hybrids delivered by a cSCK can be used as strand-displacement-activated fluorescent probes to monitor gene expression within living cells, we used iNOS as a model target system. iNOS is an important biomarker for inflammation and is greatly upregulated in response to environmental stimuli such as gamma interferon (*γ*-IFN) or lipopolysaccharide (LPS) [21, 22]. We have also previously determined a number of antisense accessible sites on iNOS mRNA that could be used as target sites by a modified reverse transcriptase random oligonucleotide library PCR method [23]. Herein we show that PNA·ODN-strand-displacement-activated fluorescence probes can be used to monitor iNOS mRNA expression in living cells by confocal microscopy following delivery by cationic shell crosslinked knedel-like nanoparticles.

## 2. Materials and Methods

### 2.1. General

The cSCK nanoparticles were prepared as previously described [24]. Anhydrous N,N-dimethylformamide (DMF), diisopropylethylamine (DIPEA), trifluoroacetic acid (TFA), meta-cresol, dichloromethane (DCM)., N-methylpyrrolidone (NMP), dimethyl sulfoxide (DMSO), and (5,6)-fluorescein-N-succinimidyl ester (FAM-NHS ester) were purchased from Sigma-Aldrich (St Louis, MO). DABCYL^plus^-N-succinimidyl ester (DABCYL^plus^-NHS) was purchased from Anaspec Inc (Fremont, CA). PNA monomers were purchased from PolyOrg Inc (Leominster, MA). Fmoc-protected amino acids were purchased from EMD chemicals (Gibbstown, NJ). 2-(1H-7-Azabenzotriazol-1-yl)-1,1,3,3-tetramethyl uronium hexafluorophosphate (HATU) was purchased from GenScript (Piscataway, NJ). Fmoc-PAL-PEG-PS resin for the solid-phase-PNA synthesis was purchased from Applied Biosystems (Carlsbad, CA). The PNAs were synthesized by solid-phase Fmoc chemistry on an Expedite 8909 DNA/PNA synthesizer on a 2 *μ*mol scale. All the oligodeoxynucleotides (ODN) and amino-modified ODNs were purchased from Integrated DNA technologies (Coralville, IA). The crude FAM-PNAs and DNA-DABCYLs were purified by a reversed-phase high-performance liquid chromatography (HPLC) on a Beckman Gold System with a UV array detector and a Varian Microsorb-MV column (C-18, 5 *μ*m, 300 Å pore size, 4.6 × 250 mm internal diameter and length). For the FAM-PNAs, a step gradient of 0–10% (2 min), 10–60% (20 min), 60–100% (20 min), and 100–0% (5 min) of solvent B (0.1% TFA in acetonitrile) in solvent A (0.1% TFA in water) was used. For the DNA-DABCYLs, a step gradient of 0% (1 min), 0–40% (5 min), 40–80% (24 min), 80–100% (3 min), and 100–0% (3 min), of solvent B (50 mM triethylammonium acetate (TEAA) in 1 : 1 water : acetonitrile) in solvent A (50 mM TEAA in water) was used. The purified PNAs and DNAs were verified by MALDI-TOF on an AppliedBiosystems 4700 mass spectrometer. The concentration of the DNAs was determined from the absorbance at 260 nm taken on a Bausch and Lomb Spectronic 1001 spectrophotometer. The concentration of the PNAs was determined from the absorbance at 260 nm at 70°C to eliminate hypochromicity due to secondary structure. For the DNAs, the molar extinction coefficient provided by the manufacturer was used. For the PNAs, the molar extinction coefficient was estimated using 13.7, 11.7, 6.6, and 8.6 mL/*μ*mol·cm for A, G, C, and T, respectively.

### 2.2. PNA-Fluorescein Synthesis and Purification

A 23-mer PNA probe antisense to the bases starting at position 480 of iNOS mRNA (FAM-iNOS-PNA) and a control probe with the same length but targeting HeLa pLuc 705 splice correction site (FAM-pLuc-PNA) were synthesized on an Expedite 8909 DNA/PNA synthesizer. After removal of the Fmoc-protecting group at the amino end of the PNA, the resin was dried with nitrogen gas and was shaken overnight with 200 *μ*L of 0.02 M FAM-NHS ester (2 eq) in DMSO, together with 2 eq DIPEA at room temperature. The resin was then washed sequentially with DMF and DCM and dried under nitrogen. The PNA was then cleaved from the support with 250 *μ*L TFA/m-cresol (4 : 1) mixture for 2–4 h. The cleavage mixture was separated from the support and the PNA precipitated by adding 1 mL cold diethyl ether and centrifuging for 10 min. The product was dried on a hot block at 55°C and dissolved in water containing 0.1% TFA. The FAM-PNAs were purified by HPLC and characterized by MALDI mass spectrometry (See Supplementary Material available online at doi:10.1155/2012/962652), UV and fluorescence spectroscopy. The overall yield for FAM-PNAs was about 5%.

### 2.3. DNA-DABCYL Synthesis and Purification

Regular and 3′-end-modified ODNs were purchased from IDT Inc. and purified by HPLC. The 17-mer DNAs modified with an amino linker at the 3′-end (50 nmol) were shaken overnight with 10 eq of DABCYL^plus^-NHS ester in 10 mM Na_2_CO_3_/NaHCO_3_ buffer (adjusted to pH 8.5 with hydrochloride acid). The products were purified by gel electrophoresis on a 20% polyacrylamide gel. Bands containing the desired product were eluted with 0.5 M ammonium acetate, 10 mM MgCl_2_, 1 mM EDTA, and 0.1% SDS, precipitated with 3 volumes of ethanol, cooled to −20°C for 30 min, and collected after centrifugation for 30 min. The DNA-DABCYLs were characterized by MALDI mass spectrometry and UV spectroscopy.

### 2.4. *In Vitro* mRNA Transcription

The PCMV-SPORT6 vector containing the iNOS mRNA gene was purchased from American Type Culture Collection (ATCC, Manassas, VA). LB media was inoculated with *E. coli* containing the vector at 37°C for 18 h after which the plasmid was isolated from the *E. coli* by using HiPure Plasmid Maxprep kit (Invitrogen). The plasmid was then digested by Xhol (Promega) to form linear DNA, which was purified by phenol extraction, ethanol precipitation and was characterized by electrophoresis on a 1% agarose gel stained with ethidium bromide. The linear DNA was then transcribed into iNOS mRNA using the RiboMAX SP6 large scale RNA transcription kit (Promega) following the manufacturer's protocol. The integrity of iNOS mRNA was verified on the 1% w/v agarose gel. All aqueous solutions used in this process were prepared with diethylpyrocarbonate- (DEPC-) treated water and the mRNA was stored at −80°C in water with 2 *μ*L (80 U) RNaseOUT recombinant RNase inhibitor (Invitrogen).

### 2.5. Displacement by Complementary DNA in Solution

 FAM-iNOS-PNA (0.2 *μ*M) and iNOS-DNA-DABCYL (0.4 *μ*M) were heated at 95°C for 3 min in a buffer containing 100 mM Tris, 5 mM MgCl_2_, and annealed at room temperature. The iNOS-DNA was then added to final concentrations of 0.1, 0.2, 0.4, and 2 *μ*M. After the fluorescence intensity reached its maximum value, each sample was then incubated for another 15 min and the fluorescent emission spectrum was collected with excitation at 488 nm. A similar procedure was followed for the FAM-pLuc-PNA·pLuc-DNA-DABCYL probe. The strand-displacement rate at 37°C was monitored by the increase in fluorescence at 525 nm as a function of time with the excitation at 488 nm.

### 2.6. Displacement by *In Vitro* Transcribed mRNA

FAM-iNOS-PNA (0.2 *μ*M) and complementary iNOS-DNA-DABCYL (0.4 *μ*M) were first annealed in 0.1 M KCl, 5 mM MgCl_2_, 10 mM Na-Hepes buffer (pH 7.11) and then *in vitro* transcribed iNOS mRNA was added to give final concentrations of 0.1, 0.2, and 0.4 *μ*M, respectively. The mixtures were heated at 65°C for 1 min and incubated at 37°C for 15 min. After the fluorescence intensity reached its maximum value, each sample was then incubated for another 15 min, and the fluorescent emission spectrum was collected with-excitation at 488 nm. All solutions were prepared with DEPC-treated dd water. A similar procedure was followed for the FAM-pLuc-PNA·pLuc-DNA-DABCYL probe. The strand-displacement kinetics were carried out in 10 mM Tris-HCl buffer (pH 7.15) by first heating the iNOS mRNA at 65°C for 1.5 min and letting cool to 37°C, after which 1 *μ*L of RNaseOUT (40 U, Invitrogen) was added. The prehybridized 1 : 2 FAM-iNOS-PNA·iNOS-DNA-DABCYL probe (1 *μ*M in PNA) was added to the mRNA solution to a final concentration of 0.05 *μ*M in the PNA and 0.025, 0.05, 0.1, and 0.25 *μ*M in iNOS mRNA. The fluorescence of the samples was monitored at 525 nm by Varian Eclipse Fluorimeter at 37°C with excitation at 488 nm as a function of time. All aqueous solutions were prepared using DEPC-treated dd water.

### 2.7. Quantitative RT PCR to Quantify iNOS mRNA Copy Numbers in RAW 264.7 Cells

RAW 264.7 cells were seeded on 10 mm Petri dish plates (Corning Inc, Lowell, MA) and grown until 70% confluence. Selected plates were then treated with 1 *μ*g/mL LPS and 300 ng/mL *γ*-IFN for 18, 6, and 0 h (without LPS and *γ*-IFN), respectively. Cells in each plate were counted with a hemocytometer and spun down in a centrifuge. Total RNA from each sample was extracted with the TRizol reagent (Invitrogen, CA) following the manufacturer's protocol and quantified by measuring UV absorbance at 260 nm. After treatment with Turbo DNase (RNase free), 0.5 *μ*g of each total RNA sample was reverse-transcribed into cDNA using SuperScript II reverse transcriptase (Invitrogen), following the manufacture's procedure. Briefly, 0.5 *μ*g of each total RNA sample was mixed with 300 ng random primers and 1 *μ*L dNTP (10 mM each) to make a solution of 12 *μ*L. The mixture was incubated at 65°C for 5 min and quickly chilled on ice. Then 4 *μ*L 5 × first-strand buffer, 2 *μ*L 0.1 M DTT, and 1 *μ*L RNaseOUT were added, and the mixture was incubated at 25°C for 2 min. Then 1 *μ*L of the SuperScript II RT was added to the mixture, incubated at 25°C for 10 min and then at 42°C for another 50 min. The reaction was inactivated at 70°C for 15 min, and the cDNA product was diluted 2500-fold for RT-PCR reaction. To generate the cDNA standard, 0.5 *μ*g mRNA prepared previously was reverse transcribed into cDNA using the same kit with exactly the same procedure. The resulting cDNA product was serially diluted by a factor of ten. The cDNAs and standards were then mixed with Power SYBR Green RT-PCR master mix (Invitrogen) and the RT-PCR was performed on a Steponeplus real-time PCR system with the following profile: 1 cycle of 50°C for 2 min, 95°C for 15 min, then 40 cycles of 95°C for 15 s, 60°C for 30 s, and 72°C for 45 s. The primers used to amplify iNOS cDNA were d(TGGTGGTGACAAGCACATTT) and d(AAGGCCAAACACAGCATACC), and for the GAPDH cDNA, the primers were d(TGGAGAAACCTGCCAAGTATG) and d(GTTGAAGTCGCAGGAGACAAC). Each well contained 25 *μ*L of reaction mixture including 2.5 *μ*L forward primer, 2.5 *μ*L reverse primer, 2.5 *μ*L double distilled water, 5 *μ*L cDNA template and 12.5 *μ*L Power SYBR Green RT-PCR master mix. The threshold cycle C_T_ was automatically set by the machine. The standard-curve method was used to determine the absolute copy number of the iNOS mRNA in cells. The comparative C_T_ (∆∆C_T_) method was used to calculate the relative increase of the iNOS mRNA level compared to the GAPDH mRNA.

### 2.8. Imaging iNOS mRNA Expression in Living Cells

RAW 264.7 cells were seeded on 10 mm glass-bottom dish (MatTek) at 5 × 10^4^ per well and incubated overnight until they reached 70% confluence. The cells were then washed with PBS and incubated in 1 mL media containing 1 *μ*g/mL LPS, 0.3 *μ*g/mL *γ*-IFN for 18 h at 37°C in a humidified atmosphere with 5% CO_2_. As a control, cells were incubated under the same conditions without LPS and *γ*-IFN. FAM-PNA·DNA-DABCYL (1 : 1.25) probes were annealed in 25 *μ*L OPTI-MEM for each sample and mixed with cSCK nanoparticles. The mixtures were incubated at room temperature for 20 min to let the cSCK associate with the probes and then were added to 75 *μ*L DMEM medium containing 10% FBS and without antibiotics. Cells were then washed with PBS and incubated with the 100 *μ*L medium containing the cSCK complexes. To maintain iNOS mRNA induction, LPS and *γ*-IFN were added again at 1 *μ*g/mL and 0.3 *μ*g/mL concentration, respectively. The final concentration of the FAM-PNA·DNA-DABCYL probes was 0.4 *μ*M in PNA, and the cSCK was 9.7 *μ*g/mL for an N/P ratio 8 : 1. After 24 h of incubation, fluorescent images of the cells were collected on a Nikon A-1 confocal microscope. The fluorescent images were processed by image J software. The mean fluorescence per cell was calculated by integrating the signal intensity of the regions of interest, then dividing by the number of cells.

## 3. Results and Discussion

### 3.1. Design and Synthesis of the Strand-Displacement Probes

**Figure 2:**
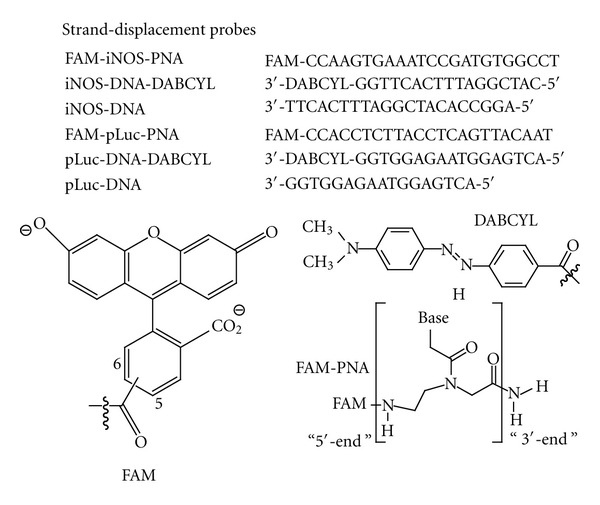
Sequences used in this study. FAM is directly linked to the amino-terminus of the PNA, while a derivative of DABCYL is linked to the 3′-terminus of the DNA through an amino linker.

The strand-displacement probes were designed to have a longer antisense PNA conjugated to the fluorophore and a shorter sense DNA conjugated to the quencher to insure that the fluorophore-bearing PNA would both kinetically and thermodynamically favor hybridization to the target mRNA (Figure 2). We chose to image iNOS mRNA because it is a biomarker for inflammation that is dramatically elevated upon treatment of cells or tissue with *γ*-interferon and LPS (lipopolysaccharide). The PNA sequence used for the construction of the fluorescent probe was selected from a number of PNAs that we had previously demonstrated to bind to *in vitro* transcribed and endogenous iNOS mRNA, and to suppress iNOS expression *in vivo* [23]. The antisense accessible sites on the iNOS mRNA were identified by an RT-ROL (reverse transcriptase-random oligonucleotide library) method that we had improved upon [25]. Transfection of selected PNA·ODN duplexes with Lipofectamine confirmed the ability of these PNAs to inhibit gene expression. *In vitro* binding assays with in vitro transcribed mRNA confirmed that a number of these sites bound both antisense ODNs and PNAs with high affinity [26]. From these, we chose the 23-mer PNA480 sequence that targets nucleotides 473–494 on iNOS mRNA. The specificity of the antisense iNOS 23-mer sequence was assessed by BLAST (basic local alignment search tool) which revealed that the next best mRNA targets were complementary to only 14 bases of the 23-mer nucleoredoxin-like, protein 1-like, and myosin VA (Myo5a) mRNAs (See Supplementary Material). The length of the quenching strand was therefore chosen to be 17 nucleotides so that the PNA·ODN duplex would be less stable than the targeted PNA·mRNA duplex, and more stable than the non-target PNA-RNA duplexes. This length would also leave a 6-nucleotide toehold for binding to the mRNA target and initiating strand displacement by branch migration.

We chose fluorescein as the fluorophore and DABCYL^plus^ as the quencher on the complementary strand as this is a common fluorophore/quencher combination [27, 28]. DABCYL^plus^ is a more soluble version of DABCYL and though its structure is proprietary, appears to involve the addition of an ethylene sulfonate chain as deduced from its molecular weight. Since it is known that a G opposite to fluorescein can also quench up to 90% of its fluorescence [29], we designed the PNA probe to have a C at the amino end (equivalent to the 5′ end of DNA), to be complementary to a G at the 3′-end of the quencher DNA strand to enhance the quenching efficiency. Because there is an A in the target iNOS mRNA at this position, we did not expect any quenching from the target mRNA. As a control, we synthesized a 23-mer PNA that is antisense to an mRNA splice correcting site in a pLuc 705 HeLa cell line which we have previously used to demonstrate the ability of cSCKs to deliver PNA*∙*DNA hybrids into this cell line. BLAST analysis indicated that there are no mRNAs sequences greater than 13 nt in mice that could activate this probe. The probes were prepared by automated solid phase Fmoc synthesis, purified by HPLC, and characterized by MALDI (See Supplementary Material).The T_m_ of the antisense and mismatched FAM-PNA·DNA-DABCYL duplexes was determined by temperature dependent fluorescence measurements to be about 68°C under physiological conditions and almost completely duplex at 37°C (See Supplementary Material).

### 3.2. Fluorescence Activation by Complementary DNA

**Figure 3:**
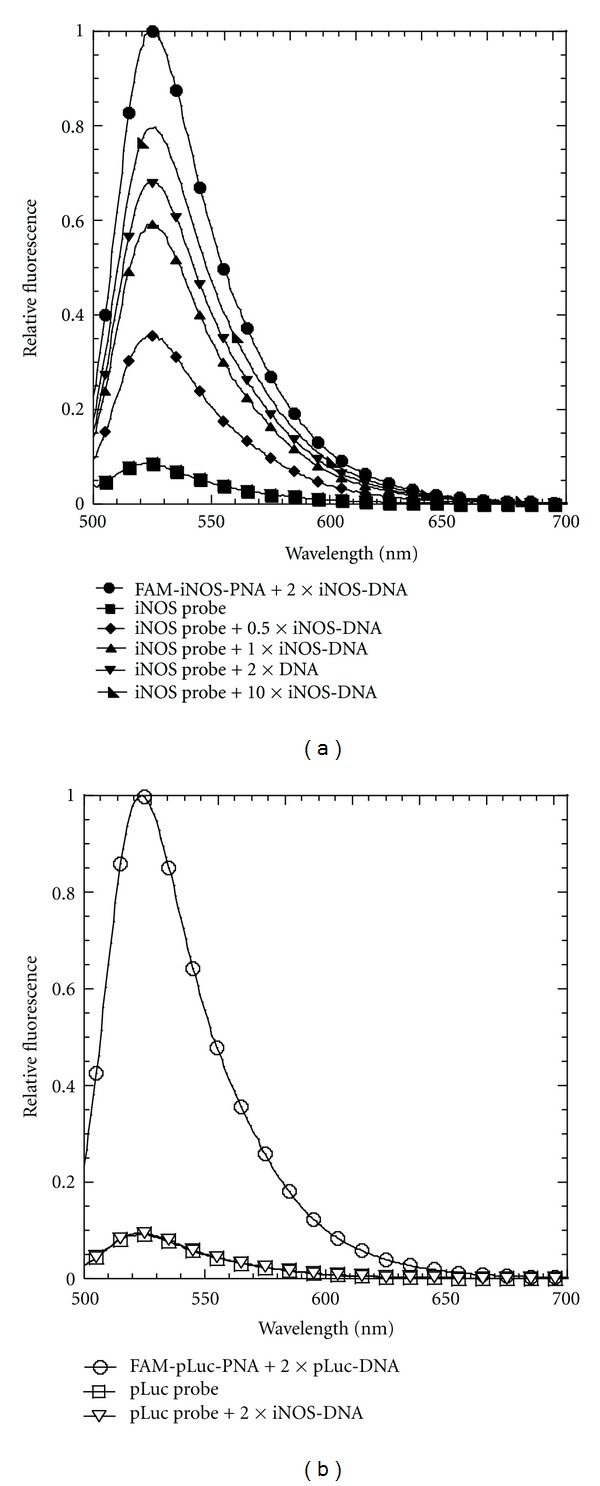
Fluorescence activation of the strand-displacement probes by DNA. (a) Fluorescence spectra of the iNOS probe (0.2 *μ*M FAM-iNOS-PNA annealed to 0.4 *μ*M iNOS-DNA-DABCYL) following addition of 0.1, 0.2, 0.4, and 2 *μ*M iNOS DNA. Positive control: 0.2 *μ*M FAM-iNOS-PNA annealed to 0.4 *μ*M iNOS-DNA. (b) Fluorescence spectrum of the pLuc probe (0.2 *μ*M FAM-pLuc-PNA annealed to 0.4 *μ*M pLuc-DNA-DABCYL) following addition of 0.4 *μ*M iNOS-DNA. Positive control: 0.2 *μ*M pLuc-FAM-PNA annealed to 0.4 *μ*M pLuc-DNA. Excitation wavelength: 488 nm, temperature: 37°C.

**Figure 4:**
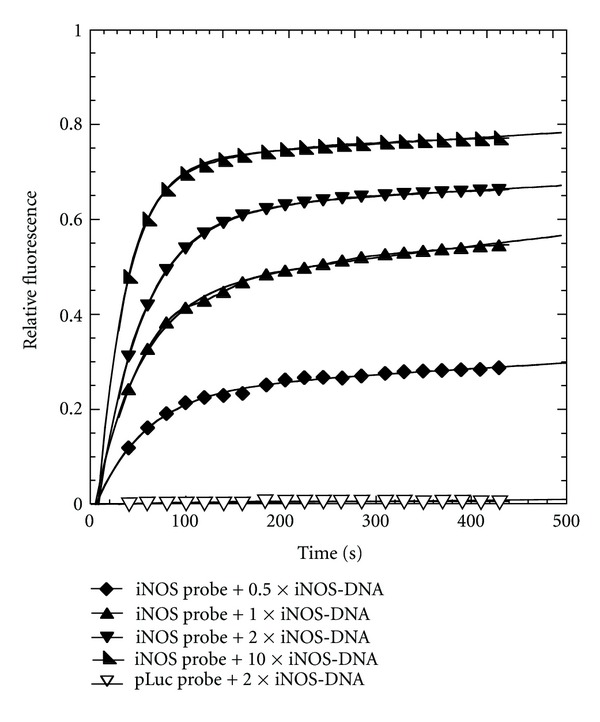
Kinetics of fluorescence activation of the strand-displacement probes by iNOS-DNA. Fluorescence emission at 525 nm of the iNOS probe (0.2 *μ*M FAM-iNOS-PNA annealed to 0.4 *μ*M iNOS-DNA-DABCYL) or the pLuc probe (0.2 *μ*M FAM-pLuc-PNA annealed to 0.4 *μ*M pLuc-DNA-DABCYL) in the presence of the indicated amount of iNOS-DNA. Excitation wavelength 488 nm. Temperature: 37°C.

The PNA*∙*DNA strand-displacement probes were first tested with a 21-mer ODN identical to the mRNA target sequence (iNOS-DNA) (Figure 3(a)). This sequence was truncated at the 3′-end to avoid introducing complementary Gs that might have quenched some of the fluorescence emission. As a positive control for the maximal amount of fluorescence achievable, FAM-iNOS-PNA was hybridized with iNOS-DNA in the absence of the iNOS-DNA-DABCYL strand. When FAM-iNOS-PNA was hybridized with a 2-fold amount of the iNOS-DNA-DABCYL in the absence of iNOS-DNA, 90% of the maximal fluorescence was quenched. Upon adding 1 equivalent of iNOS-DNA to this FAM-iNOS-PNA·iNOS-DNA-DABCYL probe (iNOS probe), almost 60% of the maximal fluorescence could be recovered. Increasing the amount of iNOS-DNA 10-fold increased the fluorescent recovery to about 80%. On the other hand, adding 2 equivalents of the iNOS-DNA to the noncomplementary FAM-pLuc-PNA·pLuc-DNA-DABCYL probe (pLuc probe) did not lead to any recovery of fluorescence (Figure 3(b)). When the strand-displacement reaction with the iNOS probe and iNOS-DNA was followed as a function of time about 80% of the maximal fluorescence was achieved in less than 10 min (Figure 4). The fluorescence recovery could be best fit to a biexponential where the major component (about 75%) occurred with a rate constant of about 0.02 s^−1^ while the slower component had a rate constant of about about 0.001 s^−1^. The origin of the slower phase is not understood at the moment. The results clearly show that the strand-displacement probe is able to effectively detect a complementary nucleic acid target in solution.

### 3.3. Fluorescence Activation by *In Vitro* Transcribed mRNA

**Figure 5:**
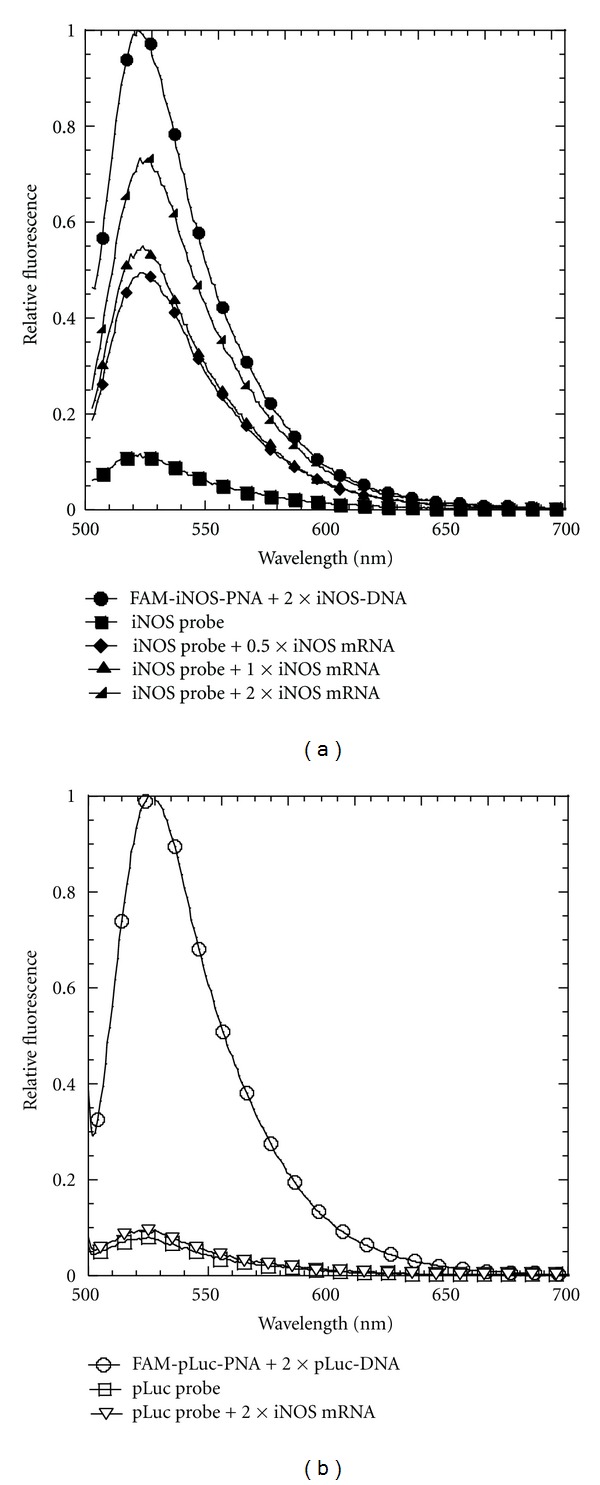
Fluorescence activation of the strand displacement probes by iNOS mRNA.(a) Fluorescence spectra of the iNOS probe (0.2 *μ*M iNOS-FAM-PNA annealed to 0.4 *μ*M iNOS-DNA-DABCYL) in the presence of 0.1, 0.2, and 0.4 *μ*M iNOS mRNA. Positive control: 0.2 *μ*M iNOS-FAM-PNA annealed to 0.4 *μ*M iNOS-DNA. (b) Fluorescence spectra of the pLuc probe (0.2 *μ*M FAM-pLuc-PNA annealed to 0.4 *μ*M pLuc-DNA-DABCYL) in the presence of 0.4 *μ*M iNOS mRNA. Excitation wavelength 488 nm. Temperature: 37°C.

Unlike the 21-mer iNOS-DNA target, *in vitro* transcribed iNOS mRNA is about 4000 nucleotides, and adopts a complicated folded structure. Studies in our lab have previously shown that the iNOS mRNA is accessible to the iNOS-PNA used for the iNOS probe [23], and that the 18-mer carboxy terminal 18-mer section, TGAAATCCGATGTGGCCT, has a high binding affinity (86 ± 26 pM) for annealed *in vitro* transcribed iNOS mRNA [26]. Also, siRNA knockdown and PNA antisense inhibition of iNOS expression suggested that the 480 site was also accessible *in vivo* [23]. The mRNA was transcribed from a cDNA clone *in vitro* and characterized by agarose gel electrophoresis (See Supplementary Material). To demonstrate that the *in vitro* transcribed iNOS mRNA has the correct sequence and could displace the quencher strand without interference from its folded structure, the iNOS probe was heated together with varying concentrations of the mRNA to 65°C for 1 min to unfold the mRNA and then cooled to 37°C for 15 min. With 0.5 to 1 equivalents of iNOS mRNA, there was about 50% recovery of fluorescence, and at 2 equivalents, about 70% demonstrating that the target mRNA sequence was indeed present and accessible after heating (Figure 5(a)). When the same procedure was carried out with the pLuc strand displacement probe no increase in fluorescence was observed, again showing the specificity of the strand displacement reaction (Figure 5(b)).

**Figure 6:**
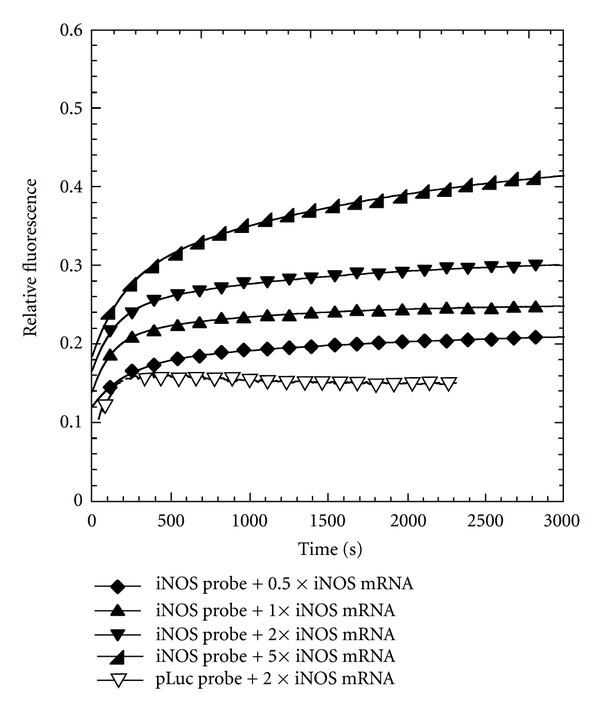
Kinetics of fluorescence activation by strand-displacement probes by iNOS mRNA. Fluorescence emission at 525 nm of the iNOS probe (0.05 *μ*M FAM-iNOS-PNA annealed to 0.1 *μ*M iNOS-DNA-DABCYL) or pLuc probe (0.05 *μ*M FAM-pLuc-PNA annealed to 0.1 *μ*M pLuc-DNA-DABCYL) with the indicated amount of iNOS mRNA. Excitation wavelength: 488 nm, temperature: 37°C.

We then investigated the ability of the probe to be activated by the full length iNOS mRNA transcript at 37°C. Initial studies with directly transcribed mRNA at 37°C were not very reproducible, so the samples were annealed first to insure that the results would be reproducible and could be correlated with independent PNA-binding measurements that were also carried out on annealed mRNA. Thus, the mRNA was first heated to 65°C for 1.5 min and then annealed at 37°C for 15 min in 10 mM Tris buffer. The iNOS probe was similarly annealed at a high concentration (1 *μ*M) and then 20-fold diluted into the mRNA solution. The fluorescence of the mixtures was monitored as a function of time and iNOS mRNA concentration at 37°C (Figure 6). When the concentration of iNOS mRNA increased from 25 nM to 250 nM, corresponding to 0.5 to 5 times the concentration of the probe, an unexpected rapid jump in fluorescence was observed, followed by an increase in the fluorescence intensity of the mixture. The pLuc probe with two equivalents of mRNA, also showed a rapid jump in fluorescence, but there was no further increase in fluorescence with time suggesting that the jump in fluorescence was due to some experimental artifact. We have not been able to establish the origin of the initial jump in fluorescence with the addition of the mRNA and it was not observed in the DNA experiment. The portion of the curve following the initial rapid rise could be fit to the same type of biexponential curve as with the DNA experiment with two approximately equal phases with rate constants of about 0.006 s^−1^ and 0.0005 s^−1^. The maximum increase in fluorescence following the rapid jump with 10-fold excess iNOS mRNA was only about 33% of that observed for a sample in which the strand displacement probe was heated and cooled with the mRNA. The lower amount of fluorescence may be due to the tertiary structure of the mRNA at 37°C which could reduce the binding affinity, and/or to the presence of multiple folded mRNAs, some of which are more kinetically accessible than others. Such folded structures, as well as protein binding, could affect the accessibility of an antisense probe *in vivo*.

### 3.4. Copy Number of iNOS mRNA in Cells

mRNAs are usually expressed at very low levels inside cells, ranging from tens to thousands of copies per cell [30]. The low copy number of mRNAs can be a problem for *in vivo* mRNA imaging because the signal generated will be very low and hard to be distinguished from background noise. So far, antisense imaging by fluorescently labeled probes are still limited to relatively abundant transcripts [2]. Normally, the expression level of iNOS is very low, but becomes greatly stimulated by LPS and *γ*-IFN, making it a good system for testing and validating antisense imaging probes. To our best knowledge, the actual copy number of iNOS mRNA inside cells before or after stimulation has not been reported. To determine the copy numbers for iNOS mRNA, we performed quantitative RT-PCR on nonstimulated RAW 264.7 cells and cells stimulated with LPS/*γ*-IFN for 6 and 18 h. We chose RAW264.7 cells for these studies because this is a mouse macrophage cell line which is well known to elevate iNOS expression in response to LPS/*γ*-IFN [31]. Furthermore, the cells primarily responsible for iNOS induction in acute lung injury (ALI) are alveolar macrophages, and we plan to ultimately extend our studies to mouse models of ALI [32]. The *in vitro* transcribed iNOS mRNA was used to generate a standard curve, and the housekeeping gene glyceraldehyde 3-phosphate dehydrogenase (GAPDH) was used as an internal control to determine the relative increase of iNOS mRNA (See Supplementary Material). Using the standard curve, the copy number for unstimulated cells was estimated to be 760 per cell, but rose 70-fold to about 53,000 after 6 h of stimulation, and 100-fold to 76,000 after eighteen hours. The ∆∆C_T_ method using GAPDH as an internal reference also showed a 96-fold increase for the iNOS mRNA after 18 h of stimulation, confirming the results obtained from the standard-curve method. The large change in copy number, and high mRNA level after stimulation makes iNOS mRNA an ideal target for development and validation of antisense imaging agents.

### 3.5. Imaging of iNOS mRNA Expression in Living Cells

**Figure 7:**
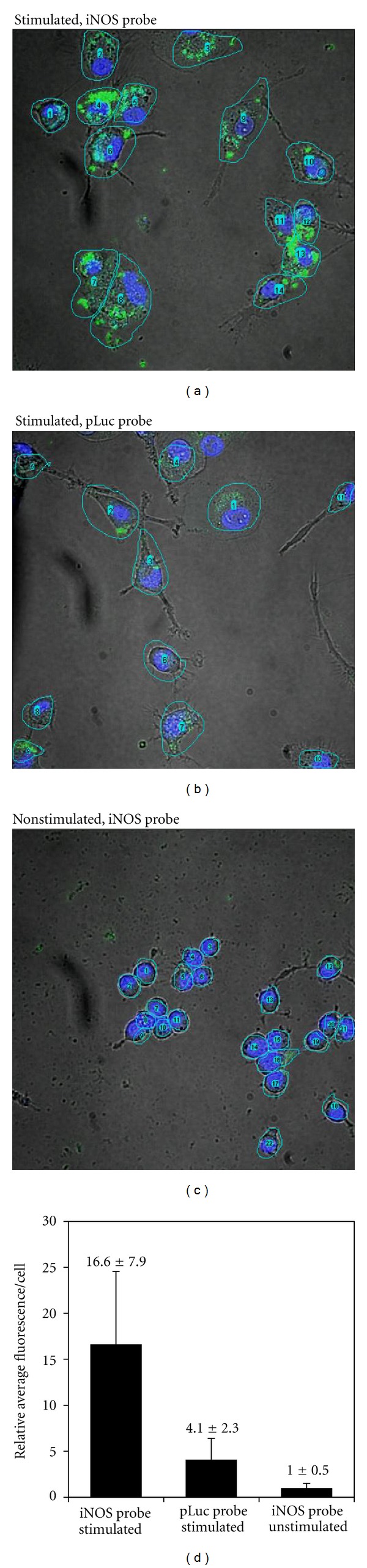
Live cell imaging of iNOS mRNA expression with the strand-displacement probes. Z-stack projection of confocal fluorescent images of RAW 264.7 cells and the quantitative analysis of fluorescence in selected regions of interests (ROIs) 24 h after transfection. The iNOS probe (0.4 *μ*M FAM-iNOS-PNA annealed to 0.5 *μ*M iNOS-DNA-DABCYL) or pLuc probe (0.4 *μ*M FAM-pLuc-PNA annealed to 0.5 *μ*M pLuc-DNA-DABCYL) was delivered with 9.7 *μ*g/mL cSCK nanoparticle at an N/P ratio of 8 : 1. Green: FAM signal. Blue: Hoechst nuclear stain.

Intracellular delivery of nucleic acids has always been a major obstacle for *in vivo* antisense imaging due to their membrane impermeability. We have found that PNAs can be efficiently delivered into cells by hybridizing the PNA with negatively charged DNA and then forming an electrostatic complex with cSCK (cationic-shell-crosslinked knedel-like nanoparticle) [19, 20, 24]. In addition to being able to form the electrostatic complex with the PNA*∙*DNA duplex, the positively charged shell of the cSCK nanoparticle also facilitates entry into cells via endocytosis, and escape of the PNA*∙*DNA duplex from the endosome by the proton sponge effect.  Figure 7 shows the results of confocal imaging of live RAW cells following with optimized concentrations of both the probes and cSCK nanoparticles. For cells treated with LPS/*γ*-IFN, and the iNOS probe, there was bright fluorescence inside the cytoplasm, indicating hybridization of the probes to the mRNA. For cells not treated with LPS/*γ*-IFN and cells treated with LPS/*γ*-IFN but with pLuc probe, there was much less observable fluorescence. Quantification of the fluorescence shows that there was a 16.6 ± 7.9-fold increase in the average fluorescence of the iNOS probes per cell that were stimulated with LPS/*γ*-IFN relative to the cells that were not stimulated, which is consistent with the expected difference in iNOS mRNA expression level. 

The average fluorescence/cell for the stimulated cells treated with pLuc probe, however, showed a 4.1 ± 2.3-fold increase in fluorescence compared to that for the iNOS probe in unstimulated cells. One possible explanation is that LPS/*γ*-IFN treatment might have caused an increased internalization of the probes which would lead to an increase in background fluorescence compared to unstimulated cells.  Figure 7 shows that stimulated cells are about two times larger in diameter than unstimulated cells which could explain the increase in background signal. LPS/*γ*-IFN stimulation may also lead to an increase in degradation rate of the probes within the cells that could increase the background signal. The same experiment was repeated one month later with similar, if not better results (See Supplementary Material). In the second experiment, a 56 ± 24-fold increase in average fluorescence per cell was observed for the iNOS probe upon stimulation, while an 8 ± 4.2-fold increase was observed for the pLuc probe. The difference in the fluorescence per cell between the iNOS and pLuc probes in the stimulated cells in the second experiment (7-fold) was also greater than that observed in the first experiment (4-fold). This second set of results, together with results from an initial experiment preceding the first experiment indicate that the results are reproducible but that there may be experiment-to-experiment variability. 

There are many other factors that could contribute to the lower-than-expected difference in fluorescence from the probes between the stimulated and unstimulated cells, such as a difference in accessibility to the targeted mRNA in stimulated and nonstimulated cells due to different protein interactions and ribosomal activity. There is also a possibility that the change in expression level of iNOS mRNA determined by RT-PCR does not properly reflect the change in expression level in the presence of nanoparticle in the cytoplasm, where the probes appear to be. We saw no fluorescence in the nucleus, either suggesting that the probes are not entering the nucleus or the mRNA is inaccessible in the nucleus. The former explanation is more likely, as unpublished experiments carried out with similar but unquenched probes do not appear to enter the nucleus. Since it has been recently reported that there can be differences in the level of a particular gene transcript in the cytoplasm and the nucleus [33], it is possible that the increase in cytoplasmic iNOS expression measured by the displacement probes is less than what is being measured by RT-PCR for the whole cell.

## 4. Conclusion

We have showed that the strand-displacement-activated PNA probes function *in vitro* and can be efficiently delivered by cSCK nanoparticles to image iNOS mRNA in living cells. The iNOS probes showed a 17-to-56-fold increase in average fluorescent signal per cell upon stimulation of cells, but the signal was only 4-to-7-fold greater than the signal seen for the noncomplementary pLuc probe. The observed increase in iNOS probe fluorescence intensity compared to unstimulated cells is much less than the expected value of about 100 determined by RT-PCR, which may be due to off target activation of the nontargeted probe, and/or activation of the nontargeted probe resulting from degradation of the quencher strand. The difference could also be due to differences in mRNA expression detected by the strand displacement probes in the cytoplasm, compared to that detected by RT-PCR in the whole cell. Nonetheless, this class of PNA-based strand-displacement probes combined with cSCK nanoparticle delivery looks promising for live-cell mRNA imaging, and merits further study and optimization. In the future, the quencher strand could be made more stable through the use of nondegradable nucleic acid analogs, and the probes shifted farther to the red for *in vivo* studies.

## Supplementary Material

BLAST results, MALDI and Tm analysis of the probes, iNOS mRNA preparation and expression level analysis by PCR, confocal imaging of iNOS expression by the probes
