# Supplementary material for: Imaging mRNA Expression in Live Cells via PNA·DNA Strand Displacement-Activated Probes
Source: J Nucleic Acids. 2012 Sep 26;2012:962652. doi: 10.1155/2012/962652 (PMC3463960; doi:10.1155/2012/962652)
Supplement: Supplementary file 1 — BLAST results, MALDI and Tm analysis of the probes, iNOS mRNA preparation and expression level analysis by PCR, confocal imaging of iNOS expression by the probes [file 962652.f1.docx]

**Supplementary Information**

Imaging mRNA Expression in Live Cells via Peptide Nucleic Acid (PNA) Strand-displacement Activated Probes

Zhenghui Wang,^1^ Ke Zhang,^1^ Karen L. Wooley,^1,2^ John-Stephen Taylor^1^

^1^Department of Chemistry, Washington University, St. Louis, MO 63130

^2^Department of Chemistry, Texas A&M University, P.O. Box 30012, College Station, TX 77842-3012

Correspondence should be addressed to John-Stephen Taylor, [taylor@wustl.edu](mailto:taylor@wustl.edu)

**Table S1.** **BLAST results for FAM-iNOS-PNA probe**

| Sequence name | Sequence complementary to PNA-iNOS-FAM probe | Number of matched base pairs |
| --- | --- | --- |
| Mus musculus nitric oxide synthase 2 (iNOS) mRNA | PNA 2 CAAGTGAAATCCGATGTGGCCT 23  \|\|\|\|\|\|\|\|\|\|\|\|\|\|\|\|\|\|\|\|\|\|  mRNA 473 GTTCACTTTAGGCTACACCGGA 494 | 22/23 |
| Mus musculus nucleoredoxin-like protein 1-like mRNA | PNA 10 ATCCGATGTGGCCT 23  \|\|\|\|\|\|\|\|\|\|\|\|\|\|  mRNA 14 TAGGCTACACCGGA 27 | 14/23 |
| Mus musculus myosin VA (Myo5a), mRNA | PNA 3 AAGTGAAATCCGAT 16  \|\|\|\|\|\|\|\|\|\|\|\|\|\|  mRNA 4632 TTCACTTTAGGCTA 4645 | 14/23 |

**Table S2. Characterization of the PNA and DNA probes**

| Probe name | Sequence | Calcd mass | Obsvd mass |
| --- | --- | --- | --- |
| FAM-iNOS-PNA | FAM-CCAAGTGAAATCCGATGTGGCCT | 6615.7 | 6620.5 |
| iNOS-DNA-DABCYL | CATCGGATTTCACTTGG-DABCYL | 5748.1 | 5748.5 |
| FAM-pLuc-PNA | FAM-CCACCTCTTACCTCAGTTACAAT | 6445.2 | 6444.5 |
| pLuc-DNA-DABCYL | ACTGAGGTAAGAGGTGG-DABCYL | 5886.2 | 5887.4 |

**Figure S1.**  **T_m_ study of FAM-iNOS-PNA•iNOS-DNA-DABCYL ( Top) and FAM-pLuc-PNA•pLuc-DNA-DABCYL (bottom).** 0.2 μM of probes were annealed in 100 mM Tris, 5 mM MgCl_2_ buffer. Fluorescence intensity of the probes was measured at excitation at 488 nm and emission at 525 nm. Ramp 1 is heating and ramp 2 is cooling and were conducted at 1°C/min. The greater hysteresis seen for FAM-iNOS-PNA•iNOS-DNA-DABCYL may be due to competing secondary structure formation due to the higher GC-content of the individual strands.


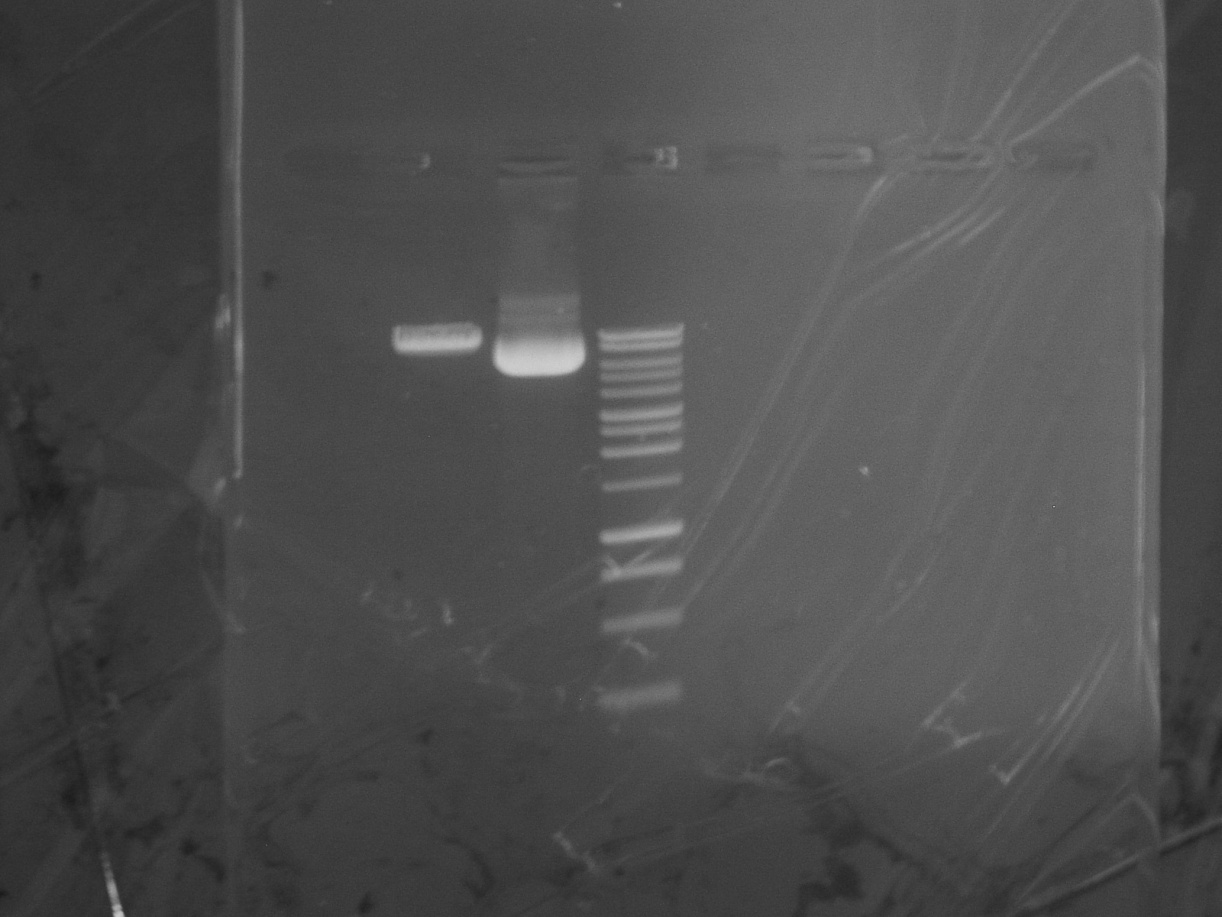


iNOS mRNA


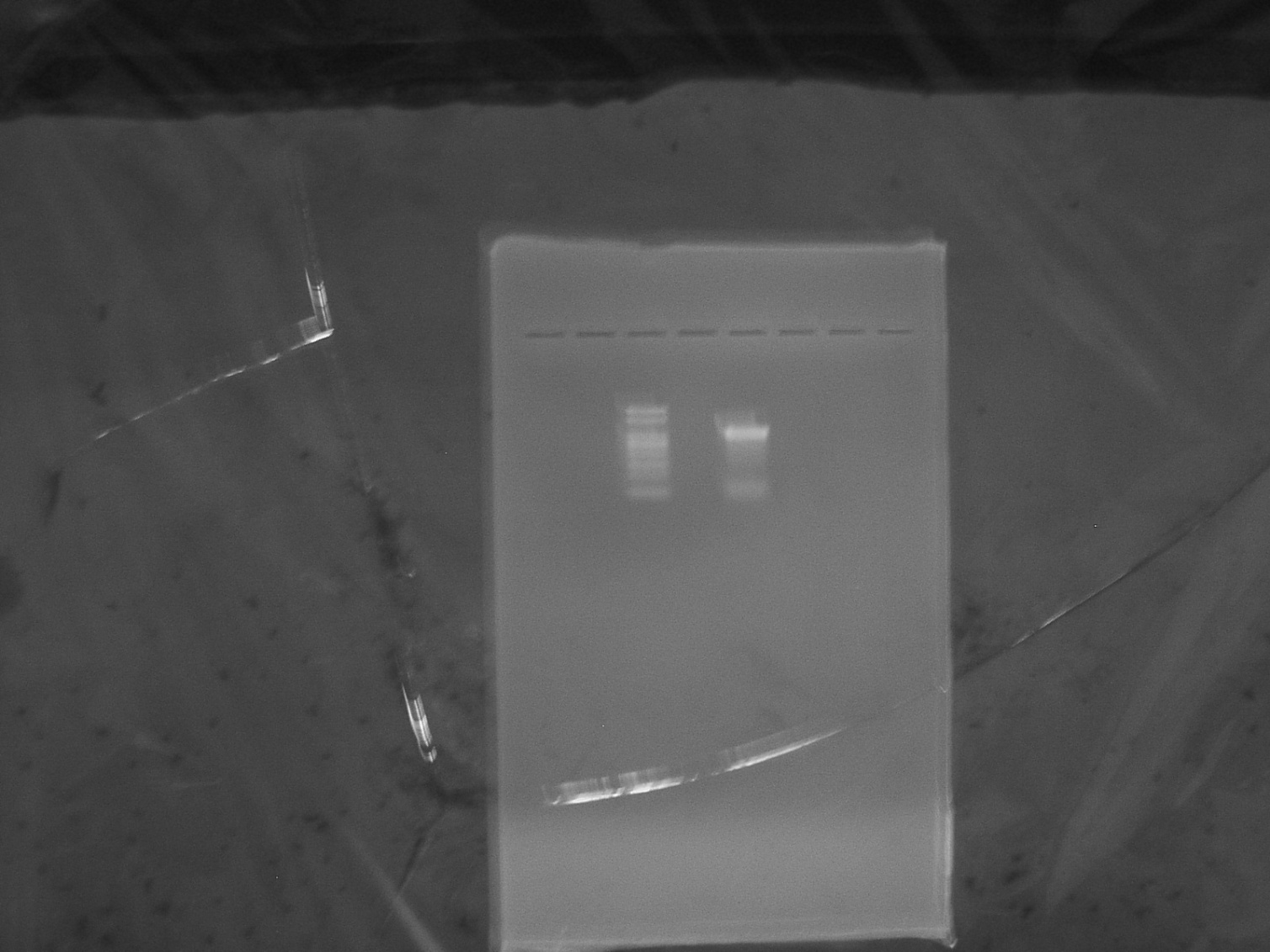


iNOS plasmid

1

2

3

4

5

**Figure S2.**  **Gel electrophoresis image of iNOS plasmid and mRNA on 1% agarose gel.** Stained with ethidium bromide. Lane 1.iNOS plasmid after enzyme digestion. 2. iNOS plasmid before enzyme digestion. 3. DNA ladder. 4. RNA ladder. 5. In vitro transcribed iNOS mRNA. The minor bands in lane 5 may be due to truncation products, or cleavage products that resulted during processing of the sample.


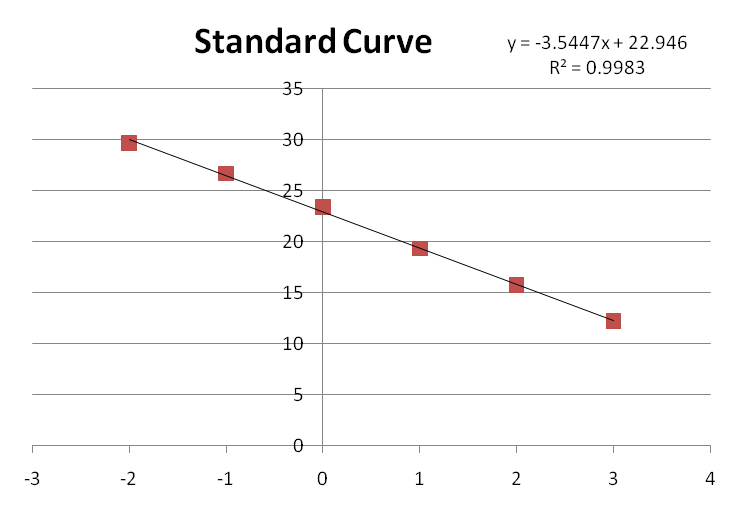
a)

| mRNA | 0.01 pg | 0.1 pg | 1 pg | 0.01 ng | 0.1 ng | 1 ng |
| --- | --- | --- | --- | --- | --- | --- |
| Log n | -2 | -1 | 0 | 1 | 2 | 3 |

b.

| **Conditions** | **C_T_** | **Copy/cell** |
| --- | --- | --- |
| Stimulated 18 h | 23.5 ± 0.1 | 76,000 |
| Stimulate 6 h | 23.8 ± 0.1 | 53,000 |
| Unstimulated | 29.5 ± 0.1 | 760 |

c.

|  | Absolute RT-PCR  (standard curve) | Relative RT-PCR  (ΔΔC_T_) |
| --- | --- | --- |
| Fold increase after 18 h | 100 | 96 |
| Fold increase after 6 h | 70 | 45 |

**Figure S3. Quantitative and relative RT-PCR to determine the absolute copy numbers of iNOS mRNA in RAW 264.7 cells.** Cells were treated with LPS and γ-IFN for 6 h or 18 h. Untreated cells were incubated under the same condition without stimuli. a) Standard curve generated from known amount of *in vitro* transcribed mRNA. b) Absolute copy number of iNOS mRNA in cells obtained from the standard curve. c) Comparison of standard curve method and ΔΔC_T_ method to determine the relative increase of iNOS mRNA in cells.

**Figure S4.** Repeat of the live cell imaging of iNOS mRNA with the strand displacement probes. Z-stack projection of confocal fluorescent images of RAW 264.7 cells and the quantitative analysis of fluorescence in selected regions of interests (ROIs). For each sample, 0.4 μM FAM-PNA∙DNA-DABCYL (1:1.25) probe was delivered with 9.7 μg/mL cSCK nanoparticles at an N/P ratio of 8:1. Green: FAM signal. Experiment was repeated one month after the experiment in Fig. 7.
